# Clinical significance of OCT4 and SOX2 protein expression in cervical cancer

**DOI:** 10.1186/s12885-015-2015-1

**Published:** 2015-12-26

**Authors:** Bo Wook Kim, Hanbyoul Cho, Chel Hun Choi, Kris Ylaya, Joon-Yong Chung, Jae-Hoon Kim, Stephen M. Hewitt

**Affiliations:** 1 Experimental Pathology Lab, Laboratory of Pathology, Center for Cancer Research, National Cancer Institute, National Institutes of Health, MSC 1500, Bethesda, MD 20892 USA; 2 Department of Obstetrics and Gynecology, Gangnam Severance Hospital, Yonsei University College of Medicine, 146-92 Dogok-Dong, Gangnam-Gu, Seoul 135-720 South Korea; 3 Department of Obstetrics and Gynecology, Samsung Medical Center, Sungkyunkwan University School of Medicine, Seoul, 135-710 Republic of Korea; 4 Department of Obstetrics and Gynecology, Kangdong Sacred Heart Hospital, Hallym University, Seoul, 135-701 South Korea

**Keywords:** Neoplastic stem cells, OCT4, SOX2, Prognosis, Survival, Uterine cervical neoplasms

## Abstract

**Background:**

Cancer stem cell markers have become a major research focus because of their relationship with radiation or chemotherapy resistance in cancer therapy. Cancer stem cell markers including OCT4 and SOX2 have been found in various solid tumors. Here, we investigate the expression and clinical significance of OCT4 and SOX2 in cervical cancer.

**Methods:**

To define the clinical significance of OCT4 and SOX2 expression, we performed immunohistochemistry for OCT4 and SOX2 on 305 normal cervical epithelium samples, 289 cervical intraepithelial neoplasia samples, and 161 cervical cancer cases and compared the data with clinicopathologic factors, including survival rates of patients with cervical cancer.

**Results:**

OCT4 and SOX2 expression was higher in cervical cancer than normal cervix (both *p* < 0.001). OCT4 overexpression was associated with lymphovascular space invasion (*p* = 0.045), whereas loss of SOX2 expression was correlated with large tumor size (*p* = 0.015). Notably, OCT4 and SOX2 were significantly co-expressed in premalignant cervical lesions, but not in malignant cervical tumor. OCT4 overexpression showed worse 5-year disease-free and overall survival rates (*p* = 0.012 and *p* = 0.021, respectively) when compared to the low-expression group, while SOX2 expression showed favorable overall survival (*p* = 0.025). Cox regression analysis showed that OCT4 was an independent risk factor (hazard ratio = 11.23, 95 % CI, 1.31 - 95.6; *p* = 0.027) for overall survival while SOX2 overexpression showed low hazard ratio for death (hazard ratio = 0.220, 95 % CI, 0.06–0.72; *p* = 0.013).

**Conclusions:**

These results suggest that OCT4 overexpression and loss of SOX2 expression are strongly associated with poor prognosis in patients with cervical cancer.

## Background

Cervical cancer is one of the most common gynecologic malignancies worldwide and remains a leading cause of cancer-related death for women in developing countries [1]. Such high mortality rates are ascribed to disease recurrence despite cervical resection as well as ineffective treatment options for advanced disease. Radiation therapy is widely employed for advanced cervical cancer, but radiation resistance is an obstacle for cancer eradication. Radiation resistance of cancer cells is acquired by intrinsic and extrinsic factors including tumor hypoxia, cell cycle and DNA repair and radiation resistance in cancer stem cells (CSCs) [2, 3].

CSCs are a small subpopulation of cancer cells that have stem cell features such as self-renewal and the ability to differentiate into multiple cell types. Radiation therapy or chemotherapy largely eliminates cancer cells, including cervical cancer, but some tumor cells survive and acquire radiation or chemotherapy resistance [4, 5]. These resistant cancer cells are difficult to eradicate, and they show properties of CSCs.

Among CSC markers, octamer-binding transcription factor 4 (OCT4) and sex determining region Y-box 2 (SOX2) are transcriptional factors involved in the regulation of several target genes including *NANOG*, *Fgf4* and *Utf1*, as well as *OCT4* and *SOX2* [6–10]. OCT4 belongs to the POU (Pit-Oct-Unc) transcriptional factor family and plays a key role in stem cell pluripotency and differentiation by determining the fate of embryonic stem cells [11]. OCT4 expression in cancer stem-like cells is associated with self-renewal and tumorigenesis via regulation of its target genes [12]. OCT4 expression has been shown to be correlated with poor tumor differentiation and metastasis, as well as poor prognosis in colon, pancreas and lung cancer [13–15]. SOX2, a member of the SRY-related HMG-box (SOX) family of transcription factors, stimulates the reprogramming of adult cells into induced pluripotent stem cells and maintains stem cell-like properties in cancer by complexing with other stem cell markers such as NANOG and OCT4 [9]. SOX2 expression was reported to be correlated with tumorigenesis, chemoresistance and maintenance of stem cell-like phenotype in cancer cells [16, 17]. In addition, SOX2 has been shown to be highly expressed in premalignant lesions such as squamous dysplasia and carcinoma in situ in lung [18]. Prior studies suggest OCT4 and SOX2 have a key role of tumorigenesis and prognosis of cancer. However, the prognostic significance of OCT4 and SOX2 is not clearly defined in cervical premalignant and malignant lesion. In this study, we investigated the clinical significance of OCT4 and SOX2 expression in cervical neoplasia.

## Methods

### Patient selection

A total of 450 patients with cervical cancer and cervical intraepithelial neoplasia (CIN) were collected from patients who enrolled at Gangnam Severance Hospital, Yonsei University College of Medicine in Seoul, Korea and the Korea Gynecologic Cancer Bank through Bio & Medical Technology Development Program of the Ministry of Education, Science and Technology, Korea between 1996 and 2010. One hundred sixty-one paraffin-embedded specimens of cervical cancer, 289 CIN and 305 matched normal tissues were included in the study. Medical records were obtained to review patient data including age, cancer stage, tumor differentiation, cell type, tumor size, lymphovascular space invasion (LVSI) and lymph node (LN) metastasis. Cervical cancer was staged according to the International Federation of Gynecology and Obstetrics (FIGO) stage and histologically classified and graded according to World Health Organization (WHO) grade. Patients with surgical indications underwent radical hysterectomy with pelvic and aortic lymph node dissection. Concurrent chemoradiation therapy was added in cases with risk factors such as LN metastasis, parametrial invasion and positive resection margin. Inoperable patients underwent radiation or chemoradiation therapy. Tissue samples and medical records were obtained with informed consent of all patients and approval of the local research ethics committee (approval no. 3-2010-0030; Seoul, South Korea). This study was additionally approved by the Office of Human Subjects Research at the National Institute of Health.

### Tissue microarray construction and immunohistochemistry

Tissue microarrays (TMAs) were constructed from 450 patients with primary invasive cervical cancer or CIN, as well as 305 matched non-adjacent normal epithelial tissues. After hematoxylin and eosin slides were reviewed by a pathologist, areas containing each category were indicated by marking them. Four 1-mm punches were then taken from the corresponding regions of the paraffin blocks and transplanted into a recipient paraffin block using a tissue arrayer (Pathology Devices, Westminster, MD).

For immunohistochemical staining, all paraffin-embedded sections were cut at 5-μm thickness followed by deparaffinization through xylene and dehydration with graded ethanols. Antigen recovery was performed in heat-activated antigen retrieval pH 6 (Dako, Carpinteria, CA) for OCT4 and SOX2, and then specimens were incubated with 3 % H_2_O_2_ for 10 min. Non-specific binding was blocked with protein block (Dako) for 20 min at room temperature. The sections were incubated with rabbit polyclonal anti-OCT4 antibodies (Abcam, Cambridge, MA; Cat. #ab19857) at 1:250 for 30 min or with rabbit monoclonal anti-SOX2 antibodies (Cell Signaling, Danvers, MA; Cat. #3579) at 1:500 for 2 h, respectively. Subsequently, antigen-antibody reaction was detected with EnVision + Dual Link System-HRP (Dako) and visualized with DAB+ (3, 3’-Diaminobenzidine; Dako). Tissue sections were lightly counterstained with hematoxylin and then examined by light microscopy. Negative controls (substitution of primary antibody with TBS) were run simultaneously. Positive controls included testicular seminoma and lung squamous cell carcinoma for OCT4 and SOX2 [19] antibodies, respectively.

### Quantitative evaluation of immunostaining

Immunohistochemically stained slides were digitized at × 20 magnification utilizing an Aperio Scanscope CS (Aperio, Vista, CA, USA). Images were reviewed using an online software application, Digital Image Hub (SlidePath, Dublin, Ireland). Once the areas were annotated, they were sent for automated image analysis utilizing TissueIA (SlidePath’s Tissue IA system, version 3.0, Dublin, Ireland). Within Tissue IA, an algorithm was developed to quantify OCT4 and SOX2 expression levels. The staining intensity of OCT4 and SOX2 was categorized as 0 (no staining), 1+ (weak), 2+ (moderate) and 3+ (strong). The overall immunohistochemical score (histoscore) was expressed as the percentage of positive cells multiplied by their staining intensity (possible range, 0–300) [20].

### Statistical analysis

Histoscores were compared using one-way ANOVA test and independent *t*-test. The immunohistochemical cut-off for high expression of tumor markers was determined through receiver operating characteristic (ROC) curve analysis. The sensitivity and (1 - specificity) for discrimination of dead and alive was determined for each immunohistochemistry (IHC) score and plotted, thus generating a ROC curve. The cut-off value was established to be the point on the ROC curve where sum of sensitivity and specificity was maximized. Kaplan-Meier survival analysis was performed to determine the association of OCT4 and SOX2 expression with survival, and the survival curves were compared between groups using log-rank tests. Multivariate analyses of hazard ratio for death were performed using Cox proportional hazards regression. Chi-square test was used to evaluate the association between OCT4 and SOX2. Statistical analyses were performed using SPSS version 21.0 (SPSS Inc., Chicago, IL). A value of *p* < 0.05 was considered statistically significant.

## Results

### Clinicopathologic characteristics of cases

Table 1 presents the patients’ clinicopathologic characteristics. Of 161 patients with cervical cancer, 118 patients were stage IIA or less and 43 patients were stage IIB or higher. The mean age was 43.3 years (range, 19–83 years). The tumor sizes ranged from 0.2 to 12.0 cm (mean, 2.8 cm). The histopathology included 131 squamous cell carcinoma, 16 adenocarcinoma, 7 adenosquamous and 7 other types (3 small cell carcinomas, 2 neuroendocrine and 2 mixed cell types). Patients with cervical cancer were evaluated for survival analysis and the mean follow-up time of surviving patients was 54.3 months (range, 1–179). Fifteen patients (9.3 %) died during the follow-up period.

**Table 1:** Patient clinicopathologic characteristics

	Frequency	%
Age	43.3^a^	
Diagnostic category		
Normal	305	40.4
Low grade CIN	59	7.8
High grade CIN	230	30.5
Cancer	161	21.3
FIGO stage		
< IIA	118	73.3
> IIB	43	26.7
Tumor differentiation^b^		
Well	2	1.3
Moderate	112	71.8
Poor	42	26.9
Cell type		
SCC	131	81.4
AD	16	9.9
Other	14	8.7
Tumor size		
≤ 4 cm	112	69.6
> 4 cm	49	30.4
LVSI^c^		
No	86	56.2
Yes	67	43.8
LN metastasis^d^		
No	115	74.2
Yes	40	25.8
HPV test in CIN^e^		
Negative	21	14.2
Positive	127	85.8

*CIN* cervical intraepithelial neoplasia, *FIGO* International Federation of Gynecology and Obstetrics, *SCC* squamous cell carcinoma, *AD* adenocarcinoma, *LVSI* lymphovascular space invasion, *LN* lymph node, *HPV* human papillomavirus

^a^mean value

^b^calculated based on 156 cases with available tumor differentiation information

^c^calculated based on 153 cases with available LV invasion information

^d^calculated based on 155 cases with available LN metastasis information

^e^calculated based on only 148 cases of CIN with available HPV test data

### OCT4 and SOX2 protein expression

**Fig. 1:**
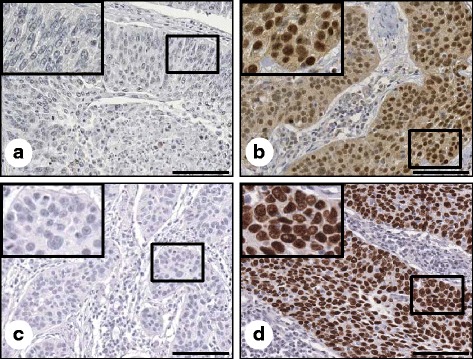
OCT4 and SOX2 expression in formalin-fixed, paraffin-embedded cervical cancer tissues. Representative immunohistochemical image of OCT4 negative (**a**) and positive (**b**), SOX2 negative (**c**) and positive (**d**). Insets show high magnification of areas indicated with boxes. Scale bar: 100 μm

Expression of OCT4 and SOX2 was evaluated by IHC in cervical neoplasia and cancer specimens. Subsequently, we performed analysis of both markers using quantitative image analysis software. Representative IHC images of OCT4 and SOX2 are presented in Fig. 1. OCT4 expression was observed primarily in the nucleus with limited cytoplasm expression, while SOX2 was restricted to the nucleus (Fig. 1). Only nuclear staining was considered OCT4- and SOX2-positive.

Of the cancer specimens, 92 of 161 cancers (57.1 %) had high expression of OCT4 (histoscore > 200) and 125 of 161 cancers (77.6 %) had high expression of SOX2 (histoscore > 30). Association of OCT4 and SOX2 expression with clinicopathologic characteristics in cervical cancer is summarized in Table 2. OCT4 and SOX2 expression was significantly different depending on diagnostic category (*p* < 0.001). OCT4 overexpression was associated with lymphovascular space invasion (*p* = 0.045), whereas loss of SOX2 expression was correlated with large tumor size (*p* = 0.015). There were no other correlations between OCT and SOX2 expression and clinicopathologic characteristics.

**Table 2:** Association between clinicopathologic characteristics and OCT4 or SOX2 expression

	OCT4		SOX2	
	Mean Histoscore (95 % CI)	*p* value	Mean Histoscore (95 % CI)	*p* value
Diagnostic category		<0.001		<0.001
Normal	113.3 (105.1–121.4)		36.5 (32.3–40.8)	
Low-grade CIN	197.6 (177.4–217.7)		40.0 (28.8–51.2)	
High-grade CIN	219.0 (211.3–226.8)		91.7 (79.9–103.5)	
Cancer	208.5 (196.7–220.3)		105.4 (91.8–119.1)	
FIGO stage		0.498		0.529
< IIA	205.7 (191.6–219.7)		108.1 (92.1–124.2)	
> IIB	215.1 (192.6–237.6)		98.3 (71.5–125.1)	
Tumor differentiation		0.438		0.112
Well + moderate	203.8 (187.7–219.8)		112.8 (94.4–131.1)	
Poor	213.4 (195.3–231.4)		90.1 (69.0–111.3)	
Cell type		0.450		0.060
SCC	205.8 (192.4–219.2)		111.7 (96.4–127.0)	
Other	217.2 (190.8–243.6)		78.3 (69.0–111.3)	
Tumor size		0.868		0.015
≤ 4 cm	208.7 (194.2–223.2)		116.5 (100.1–132.8)	
> 4 cm	206.5 (185.5–227.6)		80.3 (56.3–104.2)	
LVSI		0.045		0.106
No	195.3 (175.8–214.8)		115.5 (95.4–135.6)	
Yes	220.1 (205.3–234.9)		91.8 (71.2–112.5)	
LN metastasis		0.206		0.879
No	202.6 (187.1–218.0)		105.6 (88.6–122.7)	
Yes	221.0 (199.7–242.3)		103.1 (75.6–130.6)	
HPV test in CIN		0.292		0.907
Negative	246.8 (223.7–215.7)		124.9 (81.5–168.7)	
Positive	227.9 (215.7–240.1)		127.6 (110.5–144.6)	

*SCC* squamous cell carcinoma, *AD* adenocarcinoma, *LVSI* lymphovascular space invasion, *LN* lymph node, *HPV* human papillomavirus

We next examined the association between OCT4 and SOX2 expression, Chi-squared distribution was used in malignant and premalignant lesions. In premalignant cervical lesions, SOX2 expression presented a significant correlation with OCT4 (*p* = 0.004), while there was no association between OCT4 and SOX2 in malignant tumors (*p* = 0.543; Table 3).

**Table 3:** Association of OCT4 and SOX2 expression in CIN and cervical cancer patients

	OCT4 expression			
	No.	Low (%)	High (%)	*p* value
CIN				0.004
SOX2 Low (−)	55	29 (53.1)	26 (46.9)	
SOX2 High (+)	234	62 (26.3)	173 (73.1)	
Cancer				0.543
SOX2 Low (−)	36	15 (41.7)	21 (58.3)	
SOX2 High (+)	125	54 (43.1)	71 (56.9)	

*CIN* cervical intraepithelial neoplasia

### Prognostic significance of OCT4 and SOX2 expression

**Fig. 2:**
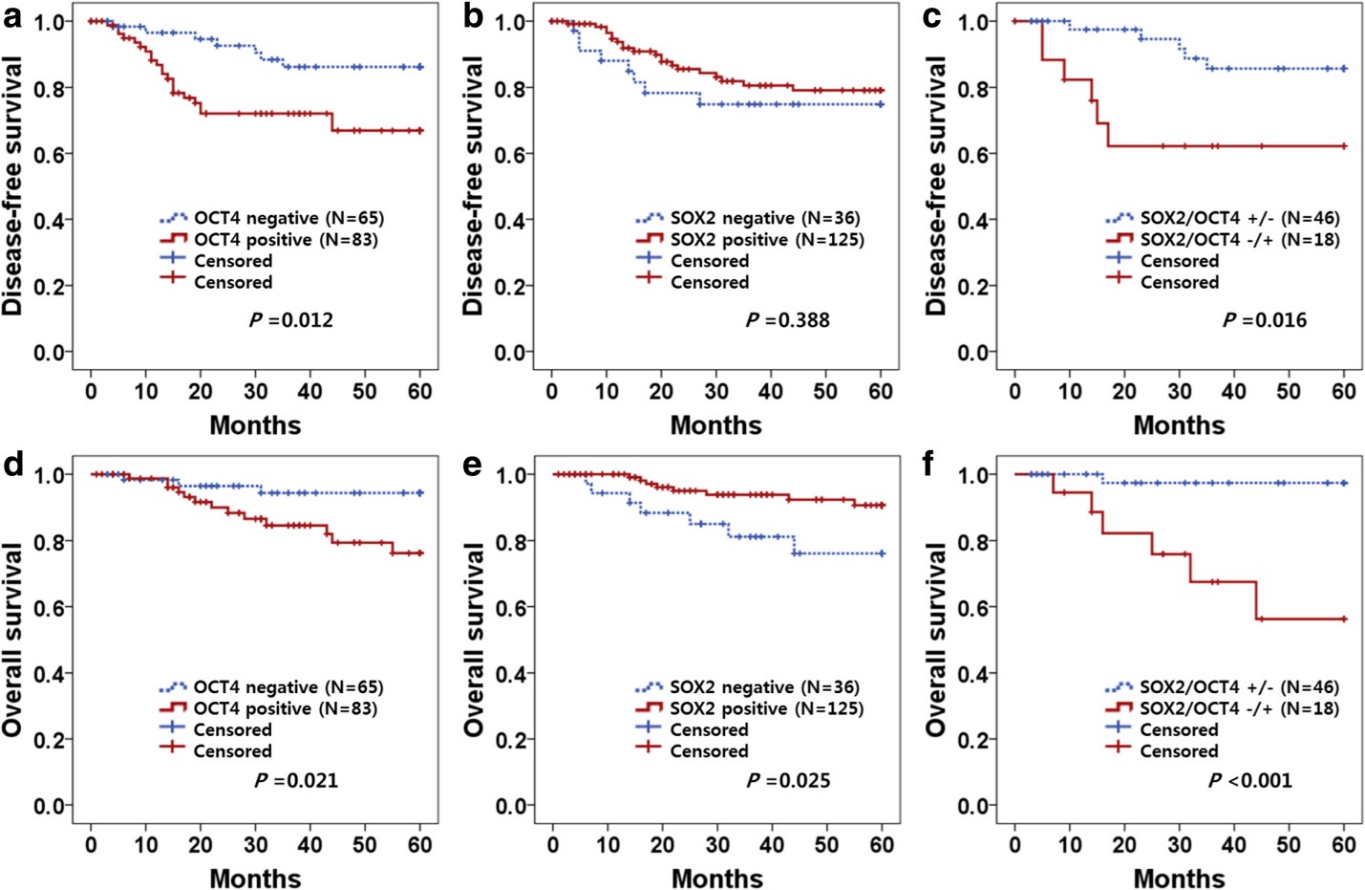
Kaplan-Meier survival curves of OCT4 and SOX2 expression in cervical cancer. Cervical cancer patients with high OCT4 expression had shorter 5-year disease-free survival (**a**, *P* = 0.012) and worse 5-year overall survival (**b**, *P* = 0.021) than those with low expression. Patients with high SOX2 expression had longer 5-year overall survival than those with low expression (**e**, *P* = 0.025). The patients with low SOX2/high OCT4 expression had shorter 5-year disease-free survival (**c**, *P* = 0.016) and worse 5-year overall survival (**f**, *P* < 0.001) than those with high SOX2/low OCT4 expression

Five-year disease-free and overall survival rates were analyzed through the Kaplan-Meier plots as shown in Fig. 2. In survival analysis with OCT4 expression, 22 recurrences and 13 deaths occurred in 83 cases of OCT4 high expression, while 7 recurrences and 3 deaths were observed in 65 cases of low expression. The 5-year disease-free and overall survival rates were 89.2 and 95.4 % in low OCT4 expression and 73.5 and 84.3 % in high OCT4 expression. OCT4 overexpression was associated with shorter disease-free and overall survival than the low expression group (*p* = 0.012 and *p* = 0.021, respectively) (Fig. 2a and d). In survival analysis of SOX2, there were 20 recurrences and 8 deaths in 125 high-expression patients, while 8 recurrences and 7 deaths occurred in 36 low-expression patients during the 5-year follow-up period. The 5-year disease-free and overall survival rates were 77.8 and 80.6 % in cases with low SOX2 expression and 84.0 and 93.6 % in case with high SOX2 expression cases. High expression of SOX2 was associated with better overall survival than low expression (*p* = 0.025) (Fig. 2e). When survival of patients with expression of high OCT4/low SOX2 was compared with survival of patients with low OCT4/high SOX2, Kaplan-Meier analysis revealed a significant difference in disease-free and overall survival (*p* = 0.016 and *p* < 0.001, respectively; Fig. 2c and f).

Cox proportional multivariate analysis of relationships between prognostic variables and survival are shown in Table 4. FIGO stage was an independent survival factor for both disease-free and overall survival analysis (*p* < 0.001 and *p* = 0.041, respectively). OCT4 overexpression showed independent poor overall survival with a hazard ratio of 11.23 (*p* = 0.027), while high expression of SOX2 presented better disease-free and overall survival compared to low expression, as shown in Table 4 (*p* = 0.019 and *p* = 0.013, respectively).

**Table 4:** Multivariate survival analysis of the association between prognostic variables and survival in cervical cancer patients

Variables	Disease-free survival		Overall survival	
	HR [95 % CI]	*P value*	HR [95 % CI]	*P value*
FIGO stage (≥ IIB)	8.67 [2.77–27.11]	<0.001	4.33 [1.06–17.72]	0.041
Tumor size (>4 cm)	1.16 [0.47–2.84]	0.739	1.79 [0.54–5.92]	0.340
LN metastasis	1.72 [0.57–5.23]	0.334	1.60 [0.40–6.41]	0.500
OCT4+	3.75 [1.24–11.55]	0.117	11.23 [1.31–95.64]	0.027
SOX2 +	0.47 [0.18–1.20]	0.019	0.22 [0.06–0.72]	0.013

*FIGO* International Federation of Gynecology and Obstetrics, *HR* hazard ratio, *LN* lymph node, *CI* confidence interval

## Discussion

OCT4 and SOX2 are important transcriptional factors involved in maintenance of pluripotency and self-renewal in cancer stem cells, aberrant expression of OCT4 and SOX2 might contribute to carcinogenesis in various cancers [15, 21, 22]. Radioresistance is important in the treatment and prognosis of cervical cancer and it is known to be associated with cancer stem cells [3]. This study examined the clinical correlation and prognostic significance of stemness-related OCT4 and SOX2 protein expression assessed by IHC in premalignant and malignant cervical tumors. The results demonstrate that OCT4 and SOX2 protein expression is elevated in premalignant and malignant cervical tumors compared to normal cervix and this finding is consistent with a previous study [23]. OCT4 was an independent poor survival factor but SOX2 showed as a favorable prognostic factor.

In this study, OCT4 protein was observed clearly in the nucleus and partially in the cytoplasm. Similar to our findings, OCT4 has been reported in the cytoplasm as well as in the nucleus in previous studies [24, 25]. This staining pattern may arise from the presence of an OCT4 isoform. OCT4 is known to have two isoforms, OCTA and OCTB. OCT4A is observed in the nucleus and OCT4B is observed in the cytoplasm in prostate and cervical cancer [24, 26]. Because OCT4 is a transcriptional regulator, the active form of OCT4 is always located in the nucleus. For this reason, we focused our automated digital image analysis on OCT4 protein expression in the nucleus only. Notably, OCT4 expression increased during cancer progression but within cancers, it was not correlated with known prognostic factors, such as stage, LN metastasis or tumor size. Nonetheless, it showed high hazard ratio of death in multivariate analysis. In previous published results, OCT4 expression was associated with unfavorable prognosis showing poor tumor differentiation, tumor invasion and metastasis in the lungs, stomach, esophagus and oral cavity [13, 25, 27, 28]. Although there have been limited reports on the association between OCT4 and prognosis in cervical cancer, Shen et al. reported that OCT4 expression was associated with radiation-resistance and unfavorable survival in locally advanced squamous cell carcinoma [29]. That study further showed OCT4 overexpression in the radiation resistance group, but it was not associated with high risk prognostic factors including FIGO stage and tumor size, which is similar to our results. It is interesting that OCT4 is associated with poor survival without correlation to known prognostic factors and even disease-free survival. As a stem cell related protein, OCT4 expression can be related more with overall survival than with disease-free survival which is possibly more related with residual tumor after resection. Further research is required to clarify the association between OCT4 and high-risk prognostic factors.

SOX2 is known to play an important role in regulating the cell cycle, DNA repair and self-renewal in stem cells [30]. It is associated with tumorigenesis, chemoresistance and maintenance of stem cell-like property in cancer cells, which suggests poor overall survival [16, 31, 32]. High expression of SOX2 was reported to be associated with a lack of cell differentiation and to contribute cell migration and invasion in cervical cancer cell line [33]. In addition, Shen et al. showed that SOX2 is highly expressed in patients with radiation resistance and predicts poor survival [29]. In contrast, SOX2 expression was associated with prolonged survival in the current study. These discrepancies might be explained by the lack of standardized methodology, different standards of interpretation or differences in studies’ patient populations. Similar to our study, Wilbertz et al. reported that SOX2 gene amplification and protein expression are associated with favorable survival outcomes in squamous cell lung cancer [34]. In addition, a recent meta-analysis reported that SOX2 expression presents a positive prognosis in non-small cell lung cancer [35]. Previously, SOX2 overexpression was reported to be associated with favorable prognosis in squamous cell lung carcinoma, but was correlated with poor survival in adenocarcinoma [34–36]. Notably, the poor survival associated with SOX2 expression that was reported in GI tract cancer mostly pertained to adenocarcinoma [37–39]. Cervical cancer consists of squamous cell carcinoma followed by adenocarcinoma and our data comprised 80.7 % squamous cell carcinoma and 14.8 % adenocarcinoma. Further research is required to clarify the prognostic significance of SOX2 in cervical cancer and variation in prognosis according to cell type.

Premalignant cervical lesion demonstrated significant correlation between OCT4 and SOX2, while malignant lesion did not present an association between OCT4 and SOX2. The lack of a correlation between OCT4 and SOX2 in malignant lesions has not been explained clearly because OCT4 and SOX2 are known to work cooperatively and self-regulate themselves via the OCT4/SOX2 complex in embryonic stem cells [6, 8]. However, in the current cancer tissue samples, OCT4 and SOX2 were associated with opposite effects on survival and lose their association in cervical cancer, as well. Similar to our results, no correlation between OCT4 and SOX2 was reported in cervical cancer [23]. In addition, Li et al. also reported that OCT4 and SOX2 were not co-expressed and also showed different survival outcomes in lung cancer tissue samples [40]. Furthermore, overexpression of SOX2 inhibited the activity of OCT4 promotor in embryonal carcinoma cells [41]. OCT4 and SOX2 are known to function cooperatively through the OCT4/SOX2 complex, but OCT4, SOX2 and Nanog have been reported to form individual complexes with nucleophosmin to control stem cell fate determination [42]. In previous study, we also observed a similar phenomenon that Nanog expression in precancerous cervical tissue was correlated with Tcl1a and pAkt but this relationship lost in cancerous tissue [43]. Considering previous results and our contradictory survival data, OCT4 and SOX2 might function independently or inhibit activity during tumor progression, and eventually lose their connection in cervical cancer.

## Conclusions

In conclusion, this study investigated the immunohistochemical expression of OCT4 and SOX2 in large number of cervical cancer patients by means of image analysis for IHC scoring. OCT4 and SOX2 showed high expression in premalignant and malignant cervical tumors. Co-expression of OCT4 and SOX2 was observed in premalignant tumors, but no association was observed in malignant cervical tumors. OCT4 high expression showed poor disease-free survival and overall survival while SOX2 high expression showed favorable overall survival in patients with cervical cancer. Cox regression analysis confirmed that OCT4, and SOX2 expression was an important prognostic indicator in cervical cancer. OCT4 was associated with poor prognosis, while SOX2 showed favorable prognosis. Our findings suggest further investigation into OCT4 and SOX2 as biomarkers in cervical cancer.

## Abbreviations

AD: adenocarcinoma
CI: confidence interval
CIN: cervical intraepithelial neoplasia
CSC: cancer stem cell
FIGO: International Federation of Gynecology and Obstetrics
H&E: hematoxylin and eosin
HPV: human papillomavirus
HR: hazard ration
IHC: immunohistochemistry
LN: lymph node
LVSI: lymphovascular space invasion
OCT-4: octamer-binding transcription factor 4: SOX2, sex determining region Y-box 2
ROC: receiver operating characteristic
SCC: squamous cell carcinoma
TMA: tissue microarray
WHO: World Health Organization
